# Psychopharmacological Prescribing Patterns Among Italian Adolescents With Anorexia Nervosa: Exploring Real‐World Treatment Practices

**DOI:** 10.1002/erv.70150

**Published:** 2026-07-08

**Authors:** Marika Orlandi, Arianna Vecchio, Giulia Andorno, Maria Pia Riccio, Cinzia Crispino, Martina Maria Mensi

**Affiliations:** ^1^ Department of Brain and Behavioral Sciences University of Pavia Pavia Italy; ^2^ Child Neurology and Psychiatry Unit IRCCS Mondino Foundation Pavia Italy; ^3^ Department of Maternal and Child Health, Child and Adolescent Neuropsychiatry AOU Federico II Napoli Italy; ^4^ Department of Translational Medical Sciences, Child and Adolescent Neuropsychiatry University of Naples Federico II Naples Italy

**Keywords:** adolescents, anorexia nervosa, benzodiazepines, child and adolescent psychiatry, eating disorders, polypharmacy, psychiatric comorbidity, psychopharmacology, psychotropic medications

## Abstract

**Objective:**

To investigate real‐world psychopharmacological prescribing patterns among Italian adolescents with anorexia nervosa (AN) compared with psychiatric controls without eating disorders, and to examine whether AN diagnosis independently predicts medication use after accounting for psychiatric comorbidities. Participants were recruited from two Italian Child and Adolescent Neuropsychiatry services to improve the representativeness of prescribing practices across different clinical settings within the Italian healthcare system.

**Method:**

This cross‐sectional cohort study included 298 female adolescents (aged 12–18 years) from 2017 to 2025. Participants were classified into AN (*n* = 149) and control (*n* = 149) groups based on DSM‐5/DSM‐5‐TR diagnoses. Sociodemographic, clinical, and pharmacological data were collected. Multivariable logistic regression models examined associations between diagnosis and prescription of any psychotropic medication, specific drug classes, and polypharmacy (≥ 2 psychotropic classes), adjusting for psychiatric comorbidities while accounting for potential site‐related differences in local prescribing practices.

**Results:**

Psychotropic medication use was more frequent among controls in unadjusted analyses. In multivariable models, AN diagnosis was not independently associated with the prescription of any psychotropic medication, antipsychotics, or antidepressants. In contrast, AN group showed a significantly higher likelihood of receiving benzodiazepines (aOR = 2.76, 95% CI 1.43–5.47) and polypharmacy (aOR = 2.37, 95%CI 1.23–4.64) compared with controls. Polypharmacy was also independently associated with psychotic disorders, personality disorders, and depressive disorders.

**Discussion:**

In this multicenter Italian real‐world cohort, psychopharmacological treatment in adolescents with AN appeared to be primarily driven by psychiatric comorbidities rather than AN diagnosis itself. However, benzodiazepine use and higher rates of polypharmacy in AN may reflect disorder‐specific clinical features and unmet treatment needs. These findings highlight the complexity of pharmacological management in adolescent AN and the need for more robust, disorder‐specific evidence to guide clinical practice.

## Key Points

- Adolescents with anorexia nervosa were more likely to receive psychopharmacological treatment than psychiatric controls, particularly antidepressants and antipsychotics.
- After adjustment for psychiatric comorbidity and recruitment site, anorexia nervosa diagnosis remained independently associated with psychopharmacological prescribing.
- Prescribing patterns appear to reflect the complexity of clinical management despite the limited evidence supporting psychotropic medications for core anorexia nervosa symptoms.
- These findings provide real‐world data that may inform future research and promote more evidence‐based prescribing practices in adolescent anorexia nervosa.

## 1 Introduction

Anorexia nervosa (AN) is a severe psychiatric disorder characterised by an intense fear of weight gain and distorted body image, leading to restrictive eating behaviours and marked cognitive‐emotional disturbances (Treasure et al. 2020).

AN most commonly develops during adolescence, with a peak age of onset around 15.5 years, and follows a prolonged course, with a mean illness duration of approximately 3.4 years (Hebebrand et al. 2024).

Adolescents with AN show high psychiatric comorbidity rates, particularly mood disorders, anxiety disorders, and obsessive‐compulsive disorders (OCD) (Attia and Walsh 2025; Carrot et al. 2017). Moreover, more than half of adolescents with AN may meet criteria for four or more comorbid diagnoses and also present suicidal ideation and self‐injurious behaviour (Wilson et al. 2026). Long‐term outcome studies indicate that only about two‐thirds of patients experience full recovery, leaving a substantial proportion of individuals with persistent symptoms or chronic illness (Hebebrand et al. 2024). Furthermore, AN is associated with a markedly increased risk of mortality: the American Academy of Paediatrics reports that the risk of premature death among patients with AN is four to five times higher than in the general population (Hornberger et al. 2021). These findings highlight the substantial clinical burden of AN and underline the need to improve treatment strategies.

Current guidelines prioritise nutritional rehabilitation and family‐based treatment (FBT) as first‐line treatments for adolescents with AN (National Institute for Health and Care Excellence 2024), and, to date, no medication has been specifically approved to treat AN.

Pharmacological interventions are therefore often used off‐label or are typically prescribed to address comorbid psychiatric symptoms rather than the AN itself. Among psychotropic medications, olanzapine, an atypical antipsychotic, is the most extensively studied drug in children and adolescents with AN (Kafantaris et al. 2011; Krauss et al. 2025; Spettigue et al. 2008). In adults with AN, it has been assigned grade B evidence for weight improvement and reduction in cognitive rigidity related to eating (Himmerich et al. 2023). Other second‐generation antipsychotics, including aripiprazole, quetiapine, and risperidone, have also been explored, but the available data are limited, and these medications are not routinely recommended (Thorey et al. 2023). Similarly, antidepressants, particularly selective serotonin reuptake inhibitors (SSRIs), have not demonstrated clear benefits in promoting weight restoration or improving core eating disorder symptoms in adolescents with AN (Balestrieri et al. 2013; Fornaro et al. 2023). Some preliminary studies suggested a potential protective effect of fluoxetine against relapse. However, these findings have not been confirmed in larger, methodologically rigorous trials (Kaye et al. 2001; Wade et al. 2015; Walsh et al. 2006).

Although pharmacological studies in adolescents with AN remain scarce and the evidence base is therefore limited, psychotropic medications are frequently prescribed in routine clinical care (Costandache et al. 2023; Himmerich et al. 2021). However, relatively little is known about real‐world prescribing patterns in this population (Couturier et al. 2019; Gorrell et al. 2020; Gowers et al. 2010; Monge et al. 2015). Available evidence is largely derived from adult or multinational samples, whereas country‐specific data in adolescents remain limited. Importantly, psychopharmacological prescribing practices may differ substantially across healthcare systems due to variations in regulatory approvals, reimbursement policies, service organisation, clinician prescribing cultures, and the availability of specialised eating disorder services (Beykloo et al. 2019). In Italy, child and adolescent psychiatric care is delivered through Child and Adolescent Neuropsychiatry services within the public healthcare system, and psychotropic prescribing in minors is additionally influenced by recommendations from the Italian Medicines Agency (Agenzia Italiana del Farmaco—AIFA). Consequently, investigating prescribing patterns within the Italian clinical context may provide clinically meaningful real‐world information regarding the pharmacological management of adolescents with AN.

In light of this, the present study aimed to investigate real‐world psychopharmacological prescribing patterns among Italian adolescents with AN compared with psychiatric controls without AN. Specifically, we examined the likelihood of receiving any psychotropic medication, specific psychotropic drug classes, and polypharmacy (defined as the prescription of two or more psychotropic drug classes), while accounting for psychiatric comorbidities. Recruitment from two Italian clinical centers in different geographic areas was intended to enhance the representativeness of prescribing practices within the Italian healthcare context rather than to conduct direct between‐site comparisons.

## 2 Methods

This cross‐sectional cohort study was conducted in accordance with the Declaration of Helsinki (World Medical Association 2013) and its subsequent amendments and received approval from the Ethics Committee of Policlinico San Matteo in Pavia, Italy (P‐20170016006) and the Ethics Committee Campania3, Italy (N.4/2025).

It included 298 help‐seeking female patients aged 12–18 years (*M* = 15.3 ± 1.42) who were admitted to two Italian Child and Adolescent Neuropsychiatry services. In Italy, Child and Adolescent Neuropsychiatry services represent the standard public healthcare services responsible for child and adolescent psychiatric care. Participants were recruited between 2017 and 2025 from one centre in northern Italy and one in southern Italy. The inclusion of two geographically distinct Italian centres was intended to increase the ecological validity and national representativeness of the sample by capturing prescribing practices across different clinical settings within the Italian public healthcare system.

We excluded individuals with intellectual disability (IQ ≤ 70) as assessed with the age‐appropriate Wechsler intelligence scale (WISC‐IV, WISC‐V, or WAIS‐IV) (Orsini et al. 2012; Orsini and Pezzuti 2015; Wechsler 2003, 2008, 2014), inadequate proficiency in Italian, and those whose parents declined to sign written informed consent or participate. All patients and caregivers provided written informed consent to participate in the study. The data were pseudonymised and are available in the Zenodo repository upon request (Mensi 2026).

Clinical, diagnostic, sociodemographic, and pharmacological data were retrospectively extracted from electronic medical records compiled during routine clinical care by trained child and adolescent neuropsychiatrists. A clinician collected sociodemographic data for each patient, including familiarity with psychiatric disorders, academic performance, social relationships, risky behaviours, and a comprehensive pharmacological history.

Academic performance was operationalised using an ordinal grading system (A–F), based on clinician‐reported school functioning and available school records. Grades reflected decreasing academic functioning: A (excellent school performance, no reported difficulties), B (minor difficulties, but satisfactory overall performance), C (moderate difficulties, reduced performance/grades), D (marked and frequent impairment), and F (severe impairment, including failing performance and/or school refusal or withdrawal from academic participation).

Social relationships were operationalised as an ordinal clinical variable reflecting the level of peer interaction and social engagement. Categories were defined as no social withdrawal (age‐appropriate peer relationships and active social engagement), moderate social withdrawal (reduced peer contact with some social interaction but evidence of avoidance or limited engagement in certain contexts), and severe social withdrawal (marked isolation or minimal peer contact), based on clinician documentation in medical records.

Risky behaviours were defined as clinically documented impulsive or self‐endangering behaviours occurring within the 6 months preceding the clinical assessment or during the index admission, as recorded in the medical records. This included both recent pre‐admission behaviours and behaviours observed during the initial treatment period when considered clinically relevant at the time of evaluation.

Medication data reflected routine clinician‐driven prescribing decisions in naturalistic inpatient and outpatient clinical practice rather than protocol‐based pharmacological interventions. Psychopharmacological data referred to medications actively prescribed at the time of clinical assessment/admission rather than lifetime pharmacological exposure.

We collected information about antipsychotics, antidepressants, benzodiazepines, mood stabilizers, and nutritional interventions. In addition to broad pharmacological classes, specific psychotropic medications were explored (olanzapine, aripiprazole, fluoxetine, and sertraline) because, despite limited evidence supporting their use in adolescents with AN, they represent the most commonly prescribed psychotropic agents in clinical practice, being approved for other psychiatric disorders in children and adolescents (Beykloo et al. 2019; Hach et al. 2026).

All participants received current psychiatric diagnoses established at admission or during the initial clinical assessment by experienced child and adolescent neuropsychiatrists according to DSM‐5 and DSM‐5‐TR criteria (American Psychiatric Association 2013, 2022), based on routine clinical evaluation and review of medical records. Participants were subsequently classified into two groups according to their diagnoses: a group of patients affected by Anorexia Nervosa or Atypical Anorexia Nervosa (AN group), and a control group (CG) of patients with other psychiatric disorders and no past or present eating disorder diagnosis.

### 2.1 Statistical Analysis

Data was analysed using RStudio (2026.01.1+403) (R Core Team 2026). Continuous variables were presented as means and standard deviations (SD), and categorical variables as frequencies and percentages. Continuous variables (age at baseline, age of onset, BMI, number of medications) were assessed for normality using Shapiro‐Wilk tests. Due to non‐normal distributions, group comparisons for continuous variables were performed with Mann–Whitney *U* tests. Categorical variables were compared between the AN and control groups using Pearson chi‐square tests.

Multivariable logistic regression models were used to compare psychopharmacological prescribing patterns between patients with AN and those in the CG. Separate models were fitted for the following outcomes: prescription of any psychotropic medication (yes/no), prescription of specific psychotropic classes (antipsychotics, antidepressants, benzodiazepines, and mood stabilizers), and polypharmacy (considering antipsychotics, antidepressants, benzodiazepines, and mood stabilizers). All models included group (AN vs. CG) as the main independent variable and were adjusted for recruiting site and psychiatric comorbid diagnoses (psychotic disorders, depressive disorders, anxiety disorders, OCD, post‐traumatic stress disorders [PTSD], sleep disorders, conduct disorders, and personality disorders). Results of logistic regression analyses are reported as adjusted odds ratios (aORs) with 95% confidence intervals (CIs) and corresponding *p*‐values. The recruitment site was included as a covariate to control for potential variability in local prescribing practices between centres, rather than to compare prescribing patterns across sites.

In the AN subgroup, logistic regression was performed to assess the association between baseline BMI (continuous) and the probability of receiving pharmacological treatment (yes/no), controlling for age. No significant association was found (*n* = 145; OR = 0.998, *p* = 0.970). Statistical significance was set at *α* = 0.05 (two‐tailed).

## 3 Results

The study included 298 female participants (*M*_age_ = 15.3 ± 1.42) divided into the AN group (*n* = 149) and the CG (*n* = 149). The groups were comparable for age, ethnicity (predominantly Caucasian), psychiatric family histories, and quality of adolescent social relationships.

Moreover, adolescents with AN were more likely to report excellent academic achievement and less likely to have poor or sufficient academic performance than those in the CG. Risky behaviours were more prevalent among CG, suggesting a more externalising profile in this subgroup. Regarding health service use, AN patients more often had prior inpatient admissions at other facilities, whereas CG more frequently reported prior emergency room (ER) accesses.

The diagnostic profile also differed between groups: psychotic disorders, depressive disorders, OCD, personality disorders, conduct disorders, and sleep disorders were unevenly distributed, with higher rates of most comorbidities in CG and more sleep disorders among AN patients.

Psychotropic medication use was common in the total sample but differed significantly by group, with CG more frequently treated and receiving a higher number of medications. Antipsychotic prescriptions differed between groups, with aripiprazole being more frequently used in CG, whereas olanzapine was more common in AN patients. Mood stabilizers and melatonin were prescribed more often in CG. Nutritional interventions were significantly more frequent in AN patients compared to CG, although not exclusive to the AN group (Table 1).

**TABLE 1:** Sociodemographic characteristics of the total sample and the two subgroups, and group comparisons.

Variable	TOT (*N* = 298)		AN (*N* = 149)		CG (*N* = 149)		*χ* ^2^/*U*	*p*
Age, *M* (SD)	15.3	(1.42)	15.3	(1.37)	15.3	(1.48)	11,306.50	0.782
Age of AN onset, *M* (SD)	—	—	13.3	(1.85)	—	—	—	—
Ethnicity, *n* (%)							6.09	0.294
Caucasian	273	(91.61)	142	(95.30)	131	(87.92)		
Asian	4	(1.34)	1	(0.67)	3	(2.01)		
African	5	(1.68)	2	(1.34)	3	(2.01)		
Latina	9	(3.02)	3	(2.01)	6	(4.03)		
Mixed	6	(2.02)	1	(0.67)	5	(3.35)		
Family history of psychiatric disorders								
Family history of psychosis	11	(3.69)	5	(3.36)	6	(4.03)	0.10	0.750
Family history of depression	99	(33.22)	48	(32.22)	51	(34.23)	0.20	0.651
Family history of anxiety	33	(11.07)	15	(10.07)	18	(12.08)	0.33	0.566
Family history of substance abuse	15	(5.03)	5	(3.36)	10	(6.71)	1.79	0.181
Family history of eating disorders	42	(14.09)	28	(18.79)	14	(9.40)	2.75	0.097
Family history of OCD	11	(3.69)	6	(4.03)	5	(3.36)	0.08	0.767
Social relations, *n* (%)							0.43	0.808
No social withdrawal	135	(45.30)	66	(44.30)	69	(46.31)		
Moderate social withdrawal	123	(41.28)	59	(39.60)	64	(42.95)		
Severe social withdrawal	24	(8.05)	10	(6.71)	14	(9.40)		
Academic performance, *n* (%)							40.75	< 0.001
A	64	(21.48)	50	(33.56)	14	(9.40)		
B	122	(40.94)	63	(42.28)	59	(39.58)		
C	54	(18.12)	12	(8.05)	42	(28.19)		
D	30	(10.07)	10	(6.71)	20	(13.42)		
F	23	(7.72)	10	(6.71)	13	(8.73)		
Risky behaviours, *n* (%)							31.40	< 0.001
No	155	(52.01)	101	(67.79)	54	(36.24)		
Non‐suicidal self‐injury (e.g., cutting)	108	(36.24)	35	(23.49)	73	(48.99)		
Drug use	4	(1.34)	1	(0.67)	3	(2.01)		
Suicidal behaviours	29	(9.73)	10	(6.71)	19	(12.75)		
Previous inpatients in other structures, *n* (%)	83	(27.85)	53	(35.57)	30	(20.13)	8.92	0.003
Previous access in the ER, *n* (%)	114	(36.89)	47	(31.54)	65	(43.62)	4.88	0.027
Diagnoses, *n* (%)								
Psychotic disorders^a^	48	(16.11)	14	(9.40)	34	(22.82)	9.93	0.002
Depressive disorders^b^	151	(50.67)	87	(58.40)	64	(42.95)	7.10	0.008
Anxiety disorders^c^	80	(26.85)	35	(23.49)	45	(30.20)	1.71	0.191
OCD^d^	19	(6.38)	14	(9.40)	5	(3.36)	4.55	0.033
PTSD^e^	15	(5.03)	8	(5.40)	7	(4.70)	0.07	0.791
Restrictive eating disorders^f^	149	(50.00)	149	(100)	0	(0)	298.0	< 0.001
Sleep disorders^g^	18	(6.04)	16	(10.74)	2	(1.34)	11.59	< 0.001
Conduct disorders^h^	8	(2.67)	1	(0.67)	7	(4.70)	4.62	0.032
Personality disorders^i^	62	(20.81)	18	(12.08)	44	(29.53)	13.77	< 0.001
BMI, *M* (SD)	—	—	15.99	(2.76)	—	—		
BMI percentiles, *n* (%)^j^								
< 3rd percentile	—	—	61	(42.1)	—	—		
3rd–10th percentile	—	—	81	(55.9)	—	—		
10th−75th percentile	—	—	3	(2.1)	—	—		
Medications (yes), *n* (%)	190	(63.76)	74	(49.66)	116	(77.85)	25.62	< 0.001
Antipsychotics	93	(31.21)	36	(24.16)	57	(38.26)	6.89	0.009
Olanzapine	30	(10.07)	23	(15.44)	7	(4.70)	9.49	0.002
Aripiprazole	48	(16.11)	9	(6.04)	39	(26.17)	22.35	< 0.001
Other antipsychotics^k^	25	(8.39)	7	(4.70)	18	(12.08)	5.28	0.022
Antidepressants	89	(29.87)	39	(26.17)	50	(33.56)	1.94	0.164
Fluoxetine	50	(16.78)	21	(14.09)	29	(19.46)	1.54	0.215
Sertraline	28	(9.40)	15	(10.07)	13	(8.73)	0.16	0.691
Other antidepressants^l^	12	(4.03)	3	(2.01)	9	(6.04)	3.126	0.077
Benzodiazepines	107	(35.91)	48	(32.21)	59	(39.60)	1.76	0.184
Mood stabilizers	25	(8.40)	4	(2.69)	21	(14.09)	12.62	< 0.001
Nutritional interventions^m^	50	(16.78)	39	(26.17)	11	(7.38)	18.84	< 0.001
Melatonin	49	(16.44)	15	(10.07)	34	(22.82)	8.82	0.003
Number of psychotropic medications^n^, *M* (SD)	1.05	(1.14)	0.85	(1.12)	1.26	(1.12)	21.14	< 0.001
0	133	(44.63)	85	(57.05)	48	(32.22)		
1	61	(20.47)	19	(12.75)	42	(28.19)		
2	65	(21.81)	29	(19.46)	36	(24.16)		
3	33	(11.07)	14	(9.40)	19	(12.75)		
4	6	(2.01)	2	(1.34)	4	(2.67)		

^a^ Psychosis, attenuated psychotic syndrome, unspecified schizophrenia spectrum and other psychotic disorder.

^b^ Major depressive disorder, dysthymia, disruptive mood dysregulation disorder.

^c^ Generalised anxiety disorder, separation anxiety.

^d^ Obsessive‐compulsive disorder.

^e^ Post‐traumatic stress disorder.

^f^ Anorexia nervosa, atypical anorexia nervosa.

^g^ Insomnia, circadian rhythm disorder.

^h^ Oppositive defiant disorder, conduct disorder.

^i^ Structuring personality disorders, since diagnosing a personality disorder in children and adolescents is not always appropriate, minors who exhibit characteristics of subthreshold personality disorders but do not fully meet the criteria of the DSM‐5 are diagnosed with structuring personality disorders.

^j^ BMI categories were assigned according to Italian BMI‐for‐age reference percentiles for girls (Cacciari et al. 2002). Underweight was defined as BMI < 3rd percentile and low weight as BMI between the 3rd and 10th percentile.

^k^ Other antipsychotics: quetiapine, risperidone, paliperidone, lurasidone, haloperidol, promazine, clozapine, clotiapine.

^l^ Other antidepressants: paroxetine, citalopram, escitalopram, venlafaxine, amitriptyline.

^m^ nutritional supplements, nasogastric tube.

^n^ Antipsychotics, antidepressants, benzodiazepine, mood stabilizers.

In multivariable logistic regression analyses examining the prescription of any psychotropic medication (Table 2), psychotropic treatment remained primarily associated with psychiatric comorbidities rather than AN status. Specifically, personality disorders, depressive disorders, psychotic disorders, and OCD were independently associated with a higher likelihood of receiving at least one psychotropic medication, while conduct disorders, sleep disorders, PTSD, and anxiety disorders were not significantly associated. AN status was not independently associated with the prescription of any psychotropic medication after adjustment for comorbid diagnoses and recruiting site.

**TABLE 2:** Multivariable logistic regression for any psychotropic medication.

Predictor	aOR (95% CI)	*p*
Personality disorders	4.08 (1.79–10.20)	0.001
Depressive disorders	3.00 (1.55–5.95)	0.001
Psychotic disorders	3.88 (1.55–11.30)	0.007
OCD	9.66 (1.79–89.60)	0.020
Conduct disorders	0.19 (0.01–1.31)	0.129
Sleep disorders	3.88 (0.45–83.70)	0.262
PTSD	1.94 (0.42–12.20)	0.431
Anxiety disorders	1.06 (0.51–2.25)	0.878
AN versus CG	1.46 (0.73–2.97)	0.292

Then, specific psychotropic classes (antipsychotics, antidepressants, benzodiazepines, and mood stabilizers) were explored. As shown in Table 3, antipsychotic prescriptions were independently associated with the presence of psychotic disorders and personality disorders. AN status and other diagnostic categories were not significantly associated with antipsychotic use after adjustment.

**TABLE 3:** Multivariable logistic regression for antipsychotic prescriptions.

Predictor	aOR (95% CI)	*p*
Psychotic disorders	5.32 (2.57–11.60)	< 0.001
Personality disorders	2.91 (1.41–6.13)	0.004
AN versus CG	1.64 (0.85–3.19)	0.141
Sleep disorders	0.39 (0.06–1.63)	0.249
OCD	2.17 (0.60–7.84)	0.229
Anxiety disorders	0.67 (0.33–1.31)	0.243
Depressive disorders	1.14 (0.61–2.14)	0.676
PTSD	1.15 (0.29–4.56)	0.841
Conduct disorders	0.34 (0.02–2.50)	0.362

Multivariable analyses of antidepressant prescriptions (Table 4) showed a significant independent association with depressive disorders and OCD. AN status and other diagnoses were not significantly associated.

**TABLE 4:** Multivariable logistic regression for antidepressant prescriptions.

Predictor	aOR (95% CI)	*p*
Depressive disorders	2.92 (1.61–5.42)	< 0.001
OCD	3.38 (1.03–11.90)	0.048
AN versus CG	1.20 (0.64–2.26)	0.563
Psychotic disorders	1.71 (0.84–3.47)	0.135
Sleep disorders	3.26 (0.50–26.40)	0.216
Conduct disorders	0.27 (0.01–2.36)	0.319
PTSD	1.68 (0.45–6.33)	0.429
Personality disorders	1.15 (0.57–2.32)	0.691
Anxiety disorders	0.95 (0.50–1.81)	0.887

In contrast to other psychotropic classes, benzodiazepine prescriptions (Table 5) were independently associated with AN status, personality disorders, depressive disorders, psychotic disorders, and OCD. Anxiety, PTSD, sleep, and conduct disorders were not significantly associated.

**TABLE 5:** Multivariable logistic regression for benzodiazepine prescriptions.

Predictor	aOR (95% CI)	*p*
Personality disorders	5.71 (2.70–12.70)	< 0.001
AN versus CG	2.76 (1.43–5.47)	0.003
Depressive disorders	2.42 (1.30–4.58)	0.006
Psychotic disorders	2.87 (1.35–6.30)	0.007
OCD	6.23 (1.48–33.80)	0.019
Conduct disorders	0.24 (0.01–2.26)	0.283
Sleep disorders	3.76 (0.21–99.30)	0.353
PTSD	1.84 (0.48–7.85)	0.381
Anxiety disorders	1.06 (0.53–2.10)	0.868

Mood stabilizer prescriptions were rare and showed no significant independent associations with any predictor (Table S1).

Finally, although polypharmacy was descriptively more common in the CG group, multivariable analysis showed that AN status was independently associated with higher odds of polypharmacy after adjustment for psychiatric comorbidities and recruiting site. Polypharmacy was also independently associated with psychotic disorders, personality disorders, and depressive disorders; OCD showed a trend towards higher odds (Table 6).

**TABLE 6:** Multivariable logistic regression for polypharmacy (≥ 2 psychotropic classes).

Predictor	aOR (95% CI)	*p*
Personality disorders	4.30 (2.07–9.28)	< 0.001
Psychotic disorders	3.99 (1.89–8.82)	< 0.001
Depressive disorders	2.13 (1.15–4.00)	0.017
AN versus CG	2.37 (1.23–4.64)	0.011
OCD	3.82 (0.98–17.10)	0.060
Sleep disorders	3.73 (0.23–95.80)	0.345
Conduct disorders	0.38 (0.02–3.10)	0.436
Anxiety disorders	1.10 (0.56–2.16)	0.783
PTSD	1.08 (0.29–4.19)	0.905

To summarise, adolescents with AN are not more likely to receive psychotropic treatment overall, but when treated, they receive more complex regimens characterised by higher benzodiazepine use and polypharmacy.

## 4 Discussion

This study investigated psychopharmacological prescribing patterns in adolescents with AN compared with controls with psychiatric disorders other than AN, focussing on cross‐sectional differences in medication use and the independent contribution of AN diagnosis after accounting for psychiatric comorbidities and recruitment site. Importantly, this study was designed to characterise prescribing practices within an Italian adolescent clinical population rather than to provide internationally representative estimates. Psychopharmacological management in AN varies substantially across healthcare systems because of differences in regulatory approvals, reimbursement systems, organisation of child and adolescent mental health services, prescribing cultures, and availability of specialised eating disorder programs (Beykloo et al. 2019; Himmerich et al. 2021). In Italy, psychotropic prescribing in minors is additionally influenced by recommendations from the AIFA. Therefore, national real‐world studies may provide clinically meaningful information even when findings are not directly generalisable across healthcare systems.

Regarding sociodemographic and clinical characteristics, adolescents with AN showed better academic functioning consistent with previous literature reporting that AN is one of the few psychiatric disorders associated with high academic achievement (Dalsgaard et al. 2020; Schilder et al. 2021), likely driven by perfectionism, a core feature of the disorder (Dahlenburg et al. 2019). Adolescents with AN also exhibited fewer externalizing features, including lower engagement in risky behaviors and reduced access to emergency services, supporting the transdiagnostic approach of AN as predominantly aligned with internalizing psychopathology (Forbush et al. 2017).

In line with these findings, the diagnostic profiles differed between groups. Adolescents with AN most frequently presented depressive disorders (58%) and anxiety disorders (23.5%). The prevalence of depressive disorders in our sample is consistent with findings from previous studies of adolescents in treatment settings using structured diagnostic assessment, ranging between 47% (Özbaran et al. 2023) and 60% (Salbach‐Andrae et al. 2008). Regarding anxiety disorders, our rate is consistent with previous literature (26%, Salbach‐Andrae et al. 2008). We also found a 10.7% prevalence of sleep disorders, which may be underestimated when relying on categorical DSM criteria, as dimensional studies report substantially higher rates (Langlet et al. 2021; Brahim et al. 2026). Although sleep disturbances are common in adolescents with AN (Burger et al. 2025; Irish et al. 2024), melatonin was prescribed less frequently in this group. This discrepancy may reflect a greater focus on core eating disorder symptoms, leading to under‐recognition of sleep disturbances as a treatment target. Sleep problems in AN have traditionally been viewed as secondary consequences of starvation and malnutrition, with clinical efforts primarily directed toward nutritional rehabilitation and weight restoration (Degasperi et al. 2024). Moreover, objective sleep findings in AN appear heterogeneous, and evidence supporting sleep‐targeted interventions, including melatonin, remains limited and has not yet resulted in clear disorder‐specific recommendations (Burger et al. 2025; Cera et al. 2026). Importantly, sleep disturbances may persist independently of weight restoration (Burger et al. 2025) and have been associated with more severe psychopathology and poorer treatment outcomes (Kim et al. 2010), suggesting that they may contribute to off‐label use of sedative medications.

On the other hand, the CG showed a broader range of psychiatric conditions, including psychotic disorders, depressive disorders, OCD, personality disorders, and conduct disorders. Adolescents with AN more frequently reported prior inpatient admissions, consistent with evidence indicating high rates of readmission in this population (Steinhausen et al. 2008), underscoring both the psychiatric severity and medical burden of the disorder.

Overall, while nutritional interventions were more common in AN, psychotropic medication use was frequent across the total sample but higher in the CG. Although adolescents with AN were less likely to receive pharmacological treatment, nearly half were nonetheless treated, likely reflecting the clinical severity of a hospital‐based sample with a high burden of comorbidities.

Regarding antipsychotic medications, olanzapine was more commonly prescribed in adolescents with AN, consistent with studies supporting its potential role in this population (Kafantaris et al. 2011; Krauss et al. 2025; Spettigue et al. 2008) and with grade B evidence for this molecule related to weight improvement and reduction in cognitive rigidity related to eating (Himmerich et al. 2023). No significant differences were observed for benzodiazepines and antidepressants. Nevertheless, aripiprazole has recently emerged as a potential alternative in selected patients with AN. Owing to its partial dopamine D2 receptor agonist activity, it has been hypothesized to improve cognitive flexibility and reduce rigid and obsessive eating‐disorder cognitions, while being associated with a lower propensity for weight gain than olanzapine (Frank 2016; Frank et al. 2017). However, evidence supporting aripiprazole in AN remains limited compared with that available for olanzapine, and its role in routine clinical practice has yet to be clearly established.

Furthermore, fluoxetine was more frequently prescribed than sertraline, consistent with both the international literature and current prescribing recommendations for adolescents.

The CG more frequently received psychotropic medications and a greater number of drug classes, likely reflecting a more heterogeneous and severe psychiatric profile, with differences particularly evident in the use of antipsychotics (especially aripiprazole) and mood stabilizers. The higher use of aripiprazole in the CG may also reflect its widespread use in child and adolescent psychiatry, according to the latest AIFA report (AIFA 2025). Aripiprazole is approved for several pediatric neuropsychiatric conditions and is frequently prescribed because of its favorable tolerability profile, including a lower risk of extrapyramidal symptoms, hyperprolactinemia, and metabolic adverse effects compared with some other second‐generation antipsychotics (Coustals et al. 2021). These characteristics may make it a preferred antipsychotic across the heterogeneous diagnostic spectrum represented in the CG.

Importantly, the prescription of psychotropic medications was primarily driven by psychiatric comorbidities rather than AN diagnosis itself. Depressive disorders, OCD, personality disorders, and psychotic disorders were all independently associated with a higher likelihood of pharmacological treatment. Prescribing patterns were consistent with clinical profiles, with antipsychotics associated with psychotic disorders and antidepressants with depressive disorders and OCD, indicating largely evidence‐based practice.

Notably, personality disorders were also associated with increased antipsychotic use. Although no medications are specifically approved for the treatment of personality disorders, pharmacotherapy is typically used off‐label as an adjunctive rather than first‐line treatment (Bateman et al. 2015). While specific subtypes were not available, cluster B disorders, particularly borderline personality disorder, may be prevalent in this age group (Klinkby et al. 2024; Sharp 2022), where antipsychotics may target impulsivity, affective dysregulation, and transient psychotic symptoms, typically for short‐term use (Bateman et al. 2015; Leichsenring et al. 2023; National Institute for Health and Care Excellence (NICE) 2009; Stoffers‐Winterling et al. 2022).

The only pharmacological class independently associated with AN status was benzodiazepines. These medications are widely used label and off‐label (Owens et al. 2010; Witek et al. 2005) and may be considered for short‐term management of acute anxiety (Himmerich et al. 2021), to manage agitation during hospitalization (Swart et al. 2011), or for pre‐meal anxiety. However, a randomized placebo‐controlled study found limited therapeutic benefit of short‐acting benzodiazepines in reducing pre‐meal anxiety in AN (Steinglass et al. 2014). However, given the limited evidence supporting benzodiazepine use in this population, along with concerns regarding dependence, co‐ingestion, and intentional misuse (Friedrich et al. 2020), this finding raises important questions about the appropriateness of their use in adolescents with AN.

Although the crude prevalence of polypharmacy was higher in the CG, multivariable analyses revealed an independent association between AN status and polypharmacy after accounting for psychiatric comorbidities. This finding suggests that, among adolescents with comparable psychiatric profiles, those with AN may be more likely to receive combinations of psychotropic drug classes, suggesting more complex pharmacological management, possibly reflecting clinical severity, treatment resistance, or the need to address multiple symptom domains, consistent with previous findings (Gorrell et al. 2020). This pattern appears coherent with other high‐risk psychiatric populations, where complex clinical presentations often lead to more intensive pharmacological strategies.

Finally, although the low BMI observed in the AN group reflects a severe clinical presentation, the degree of underweight was not independently associated with psychotropic medication use, suggesting that prescribing practices are driven more by psychiatric comorbidities rather than the degree of undernutrition.

Taken together, these results highlight that psychotropic medications are frequently prescribed in adolescents with AN despite not being first‐line interventions for core symptoms in AN. Overall, prescribing patterns appeared largely guided by psychiatric comorbidities and were broadly consistent with current clinical recommendations, with olanzapine representing the antipsychotic most commonly used in AN. Benzodiazepines were the only medication class independently associated with AN status, possibly reflecting disorder‐specific features such as eating‐related anxiety, emotional dysregulation, or sleep disturbances that may not be fully captured by categorical diagnoses (Kaye et al. 2004; Trompeter et al. 2025).

These findings are broadly consistent with emerging evidence from adult AN populations. Large‐scale studies in adults have reported high levels of psychotropic medication use and substantial exposure to multiple psychotropic classes despite the absence of approved pharmacological treatments for core eating disorder symptoms (Clausen et al. 2023). More recent evidence from severely affected adult AN populations has similarly highlighted elevated rates of psychotropic prescribing and complex pharmacological regimens, suggesting that medication use may increase with clinical severity and overall illness complexity (Zhang et al. 2026). Taken together, these data indicate that the patterns observed in adolescents may reflect a broader transdiagnostic and lifespan trend in the pharmacological management of AN, rather than a phenomenon restricted to child and adolescent services.

Nevertheless, psychopharmacological treatment in adolescents with AN remains largely based on evidence extrapolated from adult populations, with substantial off‐label use and limited controlled trials. Given the potential adverse effects of psychotropic medications, including metabolic, cardiovascular, and sedation‐related risks (Jackson et al. 2010; Lebow et al. 2013), careful risk–benefit assessment and further research in adolescent populations are needed. These findings contribute to the limited literature describing real‐world psychopharmacological management of adolescents with AN within Italian child and adolescent neuropsychiatric services.

### 4.1 Limitations

This study has some limitations. Its cross‐sectional design precludes causal inferences between clinical variables and pharmacological prescribing patterns. Second, although the inclusion of two geographically distinct Italian centres increased the representativeness of the sample within the national healthcare context, findings may not generalise to other countries or healthcare systems. Another constraint of generalisability regards the exclusion of males. Moreover, the retrospective nature of the pharmacological data did not allow for assessment of treatment timing, dosage, duration, or specific clinical indications, nor for distinguishing between continuous and as‐needed prescriptions. Additionally, psychiatric comorbidities were recorded dichotomously, without information on severity or temporal relationships with AN onset, limiting an in‐depth understanding of their role in prescribing decisions. Finally, the study captured prescribed medications rather than confirmed medication adherence or actual intake, which may be particularly problematic for interpretation in adolescent psychiatric populations, where adherence may vary substantially.

## 5 Conclusions

In conclusion, this multicenter Italian study provides real‐world evidence that psychopharmacological treatment in adolescents with AN is driven primarily by psychiatric comorbidity rather than AN diagnosis itself, with polypharmacy being more common in AN and benzodiazepines emerging as the only medication class independently associated with AN status. These findings underscore both the complexity of treating adolescent AN and the need for more robust evidence to guide pharmacological decision‐making in this population. By describing prescribing practices across two Italian child neuropsychiatric centres, these findings contribute clinically relevant national data to a literature still characterised by limited adolescent real‐world evidence.

## Funding

This research was funded by the Italian Ministry of Health (Ricerca Corrente 2025–2027). The funder did not influence the study.

## Ethics Statement

The study was conducted in accordance with the Declaration of Helsinki and was approved by the Ethics Committee of Policlinico San Matteo in Pavia, Italy (P‐20170016006) and the Ethics Committee Campania3, Italy (N.4/2025).

## Conflicts of Interest

The authors declare no conflicts of interest.

## Supporting information


**Table S1:** Multivariable logistic regression for mood stabilizer prescriptions.

## Data Availability

The original data presented in the study are openly available in Zenodo at 10.5281/zenodo.19063386 (Mensi 2026).
