# Supplementary material for: Psychopharmacological Prescribing Patterns Among Italian Adolescents With Anorexia Nervosa: Exploring Real‐World Treatment Practices
Source: Eur Eat Disord Rev. 2026 Jul 8;34(6):1626–36. doi: 10.1002/erv.70150 (PMC13656761; doi:10.1002/erv.70150)
Supplement: Supplementary file 1 [file ERV-34-1626-s001.docx]

**Table S1.** Multivariable logistic regression for mood stabilizer prescriptions

| **Predictor** | **aOR (95% CI)** | ***p*** |
| --- | --- | --- |
| RED vs CG | 0.38 (0.10–1.11) | 0.102 |
| Psychotic disorders/symptoms | 1.66 (0.59–4.34) | 0.313 |
| Personality disorders | 1.56 (0.60–3.88) | 0.344 |
| Sleep disorders | 4.47 (0.20–51.80) | 0.239 |
| Anxiety disorders | 0.93 (0.31–2.58) | 0.886 |
| Depressive disorders | 0.99 (0.39–2.52) | 0.987 |
| Conduct disorders | 0.00 (NA) | 0.996* |
| PTSD | 0.00 (NA) | 0.995* |
| OCD | 0.00 (NA) | 0.994* |

*Complete separation; OR approaching 0.
